# Improvement of Butamben Anesthetic Efficacy by the Development of Deformable Liposomes Bearing the Drug as Cyclodextrin Complex

**DOI:** 10.3390/pharmaceutics13060872

**Published:** 2021-06-12

**Authors:** Paola Mura, Francesca Maestrelli, Marzia Cirri, Giulia Nerli, Lorenzo Di Cesare Mannelli, Carla Ghelardini, Natascia Mennini

**Affiliations:** 1 Department of Chemistry, University of Florence, via Schiff 6, Sesto Fiorentino, 50019 Florence, Italy; paola.mura@unifi.it (P.M.); francesca.maestrelli@unifi.it (F.M.); marzia.cirri@unifi.it (M.C.); giulia.nerli@unifi.it (G.N.); 2 Department of Neuroscience, Psychology, Drug Research and Child Health (NEUROFARBA), Pharmacology and Toxicology Section, University of Florence, 50139 Florence, Italy; lorenzo.mannelli@unifi.it (L.D.C.M.); carla.ghelardini@unifi.it (C.G.)

**Keywords:** butamben, deformable liposomes, stearylamine, sodium cholate, double-loading, in vivo anesthetic effect

## Abstract

This work was aimed at enhancing butamben (BTB) anesthetic efficacy by the “drug-in cyclodextrin (CD)-in deformable liposomes” strategy. In the study, phase-solubility studies with natural (α-, β-, γ-) and derivative (hydroxypropyl-α-and β-, sulfobutylether-β, methyl-β) CDs evidenced the highest BTB affinity for βCD and its derivatives and indicated methyl-βCD (RAMEB) as the best carrier. Drug-RAMEB complexes were prepared by different techniques and were characterized for solid-state and dissolution properties. The best BTB–RAMEB product was chosen for entrapment in the aqueous core of deformable liposomes containing stearylamine, either alone or with sodium cholate, as edge activators. Double-loaded (DL) liposomes, bearing the lipophilic drug (0.5% *w*/*v*) in the bilayer and its hydrophilic RAMEB complex (0.5% *w*/*v*) in the aqueous core, were compared to single-loaded (SL) liposomes bearing 1% *w*/*v* plain drug in the bilayer. All vesicles showed homogeneous dimensions (i.e., below 300 nm), high deformability, and excellent entrapment efficiency. DL-liposomes were more effective than SL ones in limiting drug leakage (<5% vs. >10% after a 3 months storage at 4 °C). In vivo experiments in rabbits proved that all liposomal formulations significantly (*p* < 0.05) increased the intensity and duration of drug anesthetic action compared to its hydroalcoholic solution; however, DL liposomes were significantly (*p* < 0.05) more effective than SL ones in prolonging BTB anesthetic effect, owing to the presence of the drug-RAMEB complex in the vesicle core, acting as a reservoir. DL liposomes containing both edge activators were found to have the best performance.

## 1. Introduction

Butamben (BTB) is an ester-type local anesthetic agent utilized in topical, dermal, and mucosal formulations. Poor water solubility and short duration of action with respect to the potential duration of pain are the main drawbacks limiting its use and therapeutic efficacy. All parenteral products containing BTB have been recently removed from the market, as it was considered unsafe or not effective, probably due to its very low water solubility [1]. There was then a strong need to develop new effective topical delivery systems of BTB that are able to enhance its solubility, improve and adequately modulate its release, in turn prolonging its anesthetic effect and limiting possible systemic toxic effects.

The extensive and successful use of liposomes as safe and effective vesicular carriers for numerous drug molecules is well documented [2,3,4]. Among the different routes that can be used for their administration, liposomal formulations proved to be very effective in dermal and transdermal delivery, owing to the ability of liposomal vesicles of entrapping drugs and promoting their delivery through the skin, thus increasing their therapeutic efficacy [5,6,7,8]. In particular, it has been shown that various topical anesthetics administered as liposomal formulations actually exhibited an improved clinical efficacy in comparison with the corresponding plain drugs [9,10,11,12,13,14].

An interesting way to enhance the performance of classic liposomes, particularly in the case of poorly soluble drugs, is the entrapment of the drug in the vesicles in the form of a water-soluble complex in cyclodextrin (CD), joining into one system the relative benefits of both carriers [15]. Such a combined strategy proved to be effective not only in improving drug solubility and in avoiding its premature release from the vesicles, but also in increasing vesicle entrapment efficiency and in better modulating drug release, thus prolonging its therapeutic effect [16,17,18,19,20]. This approach was recently applied in the development of a “ropivacaine-in CD-in liposome” formulation that exhibited a more prolonged anesthetic effect compared to the classic liposomal formulation, and also a decreased drug toxicity [21].

On the other hand, elastic or deformable liposomes, also called “transfersomes”, emerged as an interesting and effective evolution of conventional liposomes, in virtue of their higher permeation and penetration ability into or through the skin [22,23]. They differ from classic liposomes due to the presence of suitable “edge activators” in the lipid bilayer, together with the phospholipid molecules; these edge activators provide elasticity and flexibility to the vesicle structure, thus allowing it to squeeze among the stratum corneum cells and more easily penetrate across the deep skin layers [24,25,26,27]. Then, further advantages in topical delivery could be obtained by applying the above cited “drug-in CD-in liposome” combined approach to elastic liposomes [28,29]. Furthermore, deformable liposomes “double-loaded” with the drug–CD complex in the aqueous phase of the vesicles and the plain drug in the bilayer generally exhibited higher entrapment efficiency and greater therapeutic effectiveness than single-loaded ones [30,31,32,33,34].

However, the selection of the most effective CD for each kind of drug is essential for maximizing the advantages of such a combined strategy. In fact, the complexing and solubilizing power of the CD towards the drug directly influences not only the vesicle entrapment efficiency but also the drug release rate from the vesicles [16,35,36]. Furthermore, the higher the stability of the drug–CD complex, i.e., the higher the affinity of the CD for the drug, the higher the vesicle stability is, due to the reduced possibility of interactions of CD molecules with the liposomal membrane components, which has been highlighted by some authors [35,37].

On the basis of all these premises and in continuation of our previous and promising studies on the effectiveness of anesthetic formulations based on the “drug-in CD-in deformable liposome” approach [30], the purpose of this work is the development of deformable liposomes bearing BTB as CD complex, aimed to enhance its therapeutic efficacy in terms of intensity and/or duration of action. Phase-solubility studies of BTB with different natural (α-, β-, and γ-) and derivative (hydroxypropyl-α-, hydroxypropyl-β-, sulfobutylether-β, methyl-β) CDs are initially performed in order to individuate the CD with the highest solubilizing and complexing ability toward the drug. Solid binary systems of BTB with the selected CD are then obtained using different techniques and are characterized for both solid-state (by DSC, XRPD, and FT-IR analyses) and dissolution properties. The best product is then chosen for the preparation of drug-double-loaded liposomal formulations, which contains stearylamine as the cationic surfactant due to its proved ability to enhance the skin penetration ability of the vesicles [30,38]. The effect on the vesicle performance of the joined use of stearylamine and sodium cholate as edge activators is also evaluated [23]. For comparison purposes, the corresponding liposomal formulations single-loaded with the whole of the plain drug in the bilayer of the vesicles are also prepared. All liposomal dispersions are characterized for particle size, zeta potential, morphology, deformability, entrapment efficiency and storage stability. Finally, their anesthetic effect is evaluated in vivo on rabbits by the conjunctival reflex text.

## 2. Materials and Methods

### 2.1. Materials

Butamben (BTB) (butyl-4-aminobenzoate), cholesterol (CH), l-α-phosphatidylcholine from egg yolk (PC), α-Cyclodextrin (αCD), γ-Cyclodextrin (γCD), stearylamine (SA) and sodium cholate (SC) were from Sigma-Aldrich (Milan, Italy). β-Cyclodextrin (βCD) and hydroxypropyl-β-cyclodextrin (HPβCD), with an average substitution degree per anhydroglucose unit of 0.6), were kindly provided by Roquette (Lille, France). Hydroxypropyl-α-cyclodextrin (HPαCD) (MS 0.6) and randomly methylated β-cyclodextrin (RAMEB), (MS 1.8) were donated by Wacker Chemie GmbH (München, Germany). Sulfobutylether-β-cyclodextrin (SBEβCD) (Captisol) was given as a kind gift from CyDex Inc. (Dallas, TX, USA). Carbopol^®^ 940 (polyacrylic acid) was kindly provided by Noveon, Inc. (Cleveland, OH, USA). All other chemicals were of analytical reagent quality.

### 2.2. Phase Solubility Studies

An excess amount of BTB was added to 10 mL of water containing growing quantities of CD in sealed vials electromagnetically stirred (750 rpm) at 25 ± 0.5 °C up to equilibrium (72 h). Aliquots were taken with a filter syringe (0.45 µm pore size) and spectrophotometrically analyzed for drug content at 287.0 nm (UV/VIS 1600 Shimadzu, Tokyo, Japan). The presence of CD did not interfere with the spectrophotometric assay of BTB. Each test was carried out in triplicate (C.V. < 3%). The apparent 1:1 binding constants of the various BTB–CD complexes were calculated from the slope of the straight lines of phase-solubility diagrams, according to the following equation [39]:

(1) $$K_{1:1}=\frac{slope}{S_{0}x\;\left(1-slope\right)}$$

### 2.3. Preparation of BTB–CD Solid Systems

Equimolar BTB–CD solid systems with the selected CD were obtained by different methods:

(a) Physical mixtures (PMs) were obtained through 15 min tumble mixing of the sieved components (75–150 µm granulometric fraction).
(b) Kneaded products (KN) were obtained by adding a small volume of an ethanol:water 50:50 *v*/*v* solution to a given amount of PM, and then were kneaded thoroughly with a pestle to obtain an homogeneous slurry; this continued until the solvent was completely removed. The obtained product was kept 24 h in an oven at 40 °C for removing traces of solvent.
(c) Coground products (GR) were prepared using a high-energy vibrational micromill (Mixer Mill MM 200 Retsch GmbH, Düsseldorf, Germany) where PMs were ball milled for 30 min at 24 Hz.
(d) Coevaporated products (COE) were obtained by coevaporation in a rotary evaporator (Heidolph Laborota 4000, Schwabach, Germany) at 55 °C of equimolar BTB–CD solutions in ethanol:water 50:50 *v*/*v*. The resulting products were kept 24 h in a vacuum desiccator to remove solvent traces.
(e) Colyophilized products (COL) were obtained by freeze-drying (Lyovac GT2, Leybold-Heraeus, Cologne, Germany) equimolar BTB–CD aqueous solutions placed in Petri dishes (20 cm diameter, 18 mm height).

Samples were stored in a desiccator and sieved (75–150 µm granulometric fraction) before use.

### 2.4. Characterization of Drug–CD Binary Systems

The solid-state properties of pure drug and CD and of the various BTB–CD solid systems were characterized by: DSC analysis (Mettler TA4000 Star^e^ system equipped with a DSC 25 cell (Mettler Toledo, Columbus, OH, USA); exactly weighed samples (5–10 mg, Mettler M3 Microbalance, Mettler Toledo, Columbus, OH, USA) put in pierced Al pans were scanned (10 °C/min) from 30 to 140 °C under static air); FT-IR spectroscopy (Perkin-Elmer Mod. 1600; Nujol dispersion in the 4000–400 cm^−1^ region, Perkin-Elmer, Waltham, MA, USA); X-ray powder diffractometry (XRPD) (Bruker D8-advance apparatus, Cu Kα radiation and graphite monochromator, 40 kV, 40 mA, 5–35° 2θ range, scan rate 0.05° s^−1^, Bruker, Billerica, MA, USA).

### 2.5. Dissolution Studies

Dissolution profiles of BTB, both alone and from its various binary systems with the selected CD, were determined according to the dispersed amount method, following the experimental conditions used in previous studies, in order to obtain comparable results [29]. Experiments were performed at 37 ± 0.5 °C by adding 600 mg of drug or drug-equivalent to 100 mL of water, in a 300 mL beaker. The medium was stirred at 100 rpm by glass three bladed propeller. At fixed intervals, aliquots were withdrawn and BTB content was assayed as described above (see Section 2.2). The same volume of fresh medium was added to keep the volume of dissolution medium constant. A correction was made to take account of the cumulative dilution. Each test was repeated three times (coefficient of variation < 5%). Dissolution behavior was characterized through the percentage of BTB dissolved at 10 min, as an index of the dissolution rate, and the dissolution efficiency at 60 min, as an index of the process overall. Dissolution efficiency (DE) was calculated according to Khan [40].

### 2.6. Preparation of Liposomes

Liposomes were prepared according to the thin layer evaporation method. The lipid phase components, put in a round-bottomed flask, were dissolved in chloroform, which was then removed by rotary evaporation under reduced pressure at 58 °C. The resulting thin film, after further drying to completely eliminate any residual solvent, was hydrated with the hydrophilic phase (10 mL water), heated for 10 min to 58 ± 1.0 °C, and then vortexed for 2 min; the treatment was repeated, performing 5 cycles in total. The resulting vesicles were then sonicated for 5 min (Sonopuls HD 2070, 300 W power, probe MS 72, Bandelin Electronic GmbH, Berlin, Germany). Drug-loaded liposomes were obtained through the following methods: (a) 1% free drug was dissolved in the lipophilic phase, and water was used as aqueous phase (single loading, SL); (b) the BTB–CD complex was dissolved in the hydrophilic phase (10 mL water) at 0.5% and the remaining 0.5 was dissolved as such in the lipophilic phase (double-loading, DL).

### 2.7. Characterization of Liposomes

The mean diameter, polydispersity index (PDI) and zeta potential of freshly prepared vesicles were determined by Photon Correlation Spectroscopy (PCS) using a ZetaSizer Nano ZS90 Malvern Instruments Ltd., Malvern, UK) set at 25 ± 0.1 °C after proper dilution with distilled water to prevent multiscattering phenomena. For each dispersion, six independent samples were collected, each of which was analyzed four times. The average size distribution was then determined, referring to the mode, which is the value best approximating the vesicle mean diameter. For vesicle charge determination, each liposomal dispersion, suitably diluted with distilled water, was dropped into the ZetaSizer electrophoretic cell, and the zeta potential was determined by electrophoretic mobility measurement. Each sample was measured six times at 25 ± 0.1 °C.

Morphological examination of the liposomal dispersions was performed by transmission electron microscopy (Philips, TEM CM 12, Andover, MA, USA) and scanning electron microscopy (FIB-SEM Gaia 3, Tescan s.r.o., Brno, Czech Republic). A drop of sample was stained with 2% *w*/*v* phosphotungstic acid solution and placed on copper grids with carbon films for viewing.

Entrapment efficiency (EE%) of loaded liposomes was indirectly determined by separation of the free drug from the vesicles by the dialysis method. Following a previously developed procedure [12], a sample (3 mL) of drug-loaded liposomal dispersion was put into a cellulose acetate dialysis bag (Spectra/Por^®^, MW cut-off 12,000, Spectrum, Mississauga, ON, Canada), which was sealed and immersed into a closed vessel with 150 mL of distilled water at 20 °C, magnetically stirred at 30 rpm. Samples of the receiving medium, withdrawn at time intervals, were replaced with an equal volume of fresh solvent and spectrometrically assayed for drug content, as described above. The experiment was stopped when constant drug concentrations were obtained in subsequent withdrawals. The percentage of encapsulation efficiency (EE%) was calculated according to this equation:

(2) $$EE\%=\frac{\left[total.drug\right]-\left[diffused.drug\right]}{\left[total.drug\right]}$$

Three separate experiments were performed and the results are given as the mean ± SD.

Elasticity of vesicles was assessed by a LiposoFast-Basic membrane extruder (Avestin GmbH, Mannheim, Germany) connected to a 3 atm pressure source. The vesicles size was determined as a mean of five experiments, before and after 11 times extrusion through a 100 nm pore size nitrocellulose membranes (Isopore, Millipore, Bedford, MA, USA). The results are the mean.

### 2.8. Stability Studies

Stability of drug-loaded liposomes was checked for 3 months. The vesicle dispersions were kept at 4 ± 1 °C, and, at fixed time intervals, they were examined for size, polydispersity, and zeta potential. Drug leakage at the end of the storage period was also determined.

### 2.9. Gel Preparation

Liposomal dispersions were formulated as Carbopol gel and tested for in vitro drug permeation properties and in vivo drug anesthetic effect. For gel base preparation, 0.5 g Carbopol 940 was added to 99.5 mL of bidistilled water, under constant stirring for 24 h at room temperature; gelation was then achieved by triethanolamine addition up to neutral pH. The gel was kept in closed containers, at 4 °C, away for the light. Drug loaded-gels were obtained by a previously reported technique [12]. Briefly, the Carbopol gel base was carefully mixed (50/50 *w*/*w* ratio) with 1% BTB as liposomal suspension or as simple hydroalcoholic solution (used as reference), obtaining in all cases a final drug concentration in the gel of 0.5% (*w*/*w*).

### 2.10. In Vitro Permeation Studies through Excised Animal Membrane

In vitro permeation studies were carried out using vertical Franz diffusion cells (Rofarma, Gaggiano, Italy) and rabbit ear skin (obtained from a local slaughterhouse) as a percutaneous absorption model, according to a previously developed method [29]. Briefly, the skin, after depilation, was excised from the connective tissue, washed with water, gently dried with a filter paper and then preserved at −25 °C. Before use, the skin was thawed, prehydrated for 1 h with pH 7.4 phosphate buffer solution, and then placed in the diffusion cell, with the stratum corneum side facing the donor chamber, and the dermal side the acceptor medium (14.5 mL of pH 7.4 phosphate buffer solution, thermostated at 37 ± 0.5 °C and kept under magnetic stirring at 50 rpm [29]). The donor compartment contained 0.15 g of liposomal dispersion or 0.3 g of gel. At predetermined intervals, 0.5 mL samples were collected from the acceptor compartment and replaced with equal volumes of fresh acceptor medium. The drug content was spectrophotometrically determined as described above (see Section 2.2), and the concentration was corrected for the cumulative dilution. The drug permeability coefficients (Kp, cm/h) were determined according to this equation:

Kp = J/Cd(3)

where J is the drug flux through the skin (mg/h.cm^2^), calculated from the linear portion of the cumulative amount of drug permeated per unit area versus time plots, and Cd the initial drug concentration in the donor compartment. Results were expressed as means ± standard deviation (*n* = 6 independent samples).

### 2.11. In Vivo Studies

The anesthetic effect of BTB formulated in aqueous Carbopol gel, as hydroalcoholic solution, due to its very poor water solubility (reference formulation), or loaded in liposomes, was tested in albino rabbits through the conjunctival reflex test [30,31]. Male albino rabbits weighing 2.5–3.0 kg (Morini, San Polo d’Enza, Italy) were utilized. A single rabbit was housed per cage and placed in the experimental room 24 h before the test, for acclimatization. Rabbits were kept at 23 ± 1 °C with a 12 h light/dark cycle, under standard diet regimen and free access to water. All studies were in agreement with the Directive 2010/63/EU of the European Parliament and of the European Union Council (22 September 2010) on the protection of animals utilized for scientific purposes. The ethical policy of Florence University follows the Guide for Care and Use of Laboratory Animals of U.S. National Institutes of Health (NIH Publication No. 85–23, revised 1996; Florence University assurance number: A5278-01). The Animal Subjects Review Board of Florence University (A3678, 2017) approved the experiments, which were carried out according to ARRIVE guidelines [41]. Everything has been done to limit animal suffering and the number of animals used. For each formulation, a group of six rabbits was used. A constant dose of each sample was instilled in the conjunctival sac of the rabbit left eye, while an analogous placebo formulation was instilled in the right eye as control. A cat whisker was used at suitable times to cause the conjunctival reflex. The intensity of the drug anesthetic action is directly related to the number of stimuli required to induce the reflex.

### 2.12. Statistical Analysis

ANOVA coupled with the Student–Newman–Keuls multiple comparison post-test (GraphPad Prism, Version 6, GraphPad Prism Software, San Diego, CA, USA) was used for statistical analysis of data. Differences with a *p*-value less than 0.05 were considered statistically significant.

## 3. Results and Discussion

### 3.1. Phase-Solubility Studies

The proper choice of the most suitable CD to use for drug complexation is critical both to exploit at the best the benefits of the “drug-in CD-in liposome” strategy and to avoid possible problems of vesicle destabilization due to the CD presence [16,35,36,37]. Then, as a first step of this work, phase-solubility studies of BTB with a series of natural and derivative CDs were performed.

**Figure 1:**
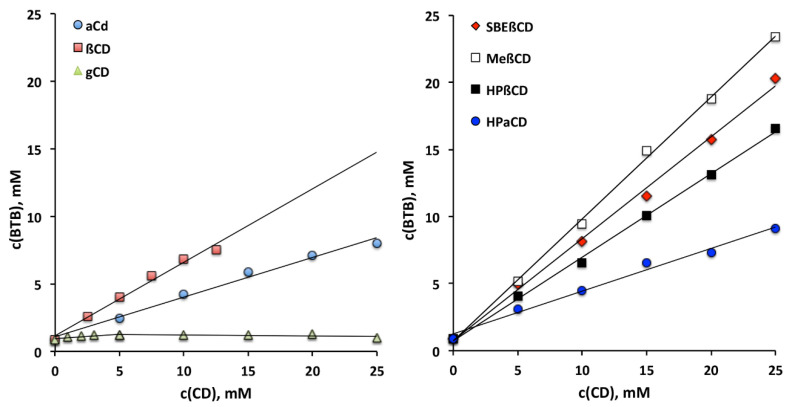
Phase-solubility diagrams of butamben (BTB) in the presence of natural (left) or derivative (right) CDs in water at 25 ± 0.5 °C.

The results of these studies (Figure 1) showed that the solubility of BTB increased linearly with increasing the concentration of the examined CDs, exhibiting A_L_-type phase-solubility diagrams, indicative of the formation of highly-soluble complexes of presumed 1:1 stoichiometry, with the only exception being γCD, where a B_S_-type phase-solubility diagram was instead observed, an index of the formation of a poorly soluble complex [39]. The apparent 1:1 stability constant of the complexes and the solubilizing efficiency values of the different CDs towards BTB are collected in Table 1.

As it appears evident, among the natural CDs, βCD emerged as the most effective complexing and solubilizing agent for BTB, despite its lower water solubility, suggesting that its cavity has the most suitable size to host the drug molecule. This result was confirmed also in the case of the examined CD derivatives, where HPβCD was a more effective partner than HPαCD. Moreover, it can be observed that the stability constants of the complexes with all the examined βCD-derivatives were distinctly higher than that of the parent βCD, suggesting that the presence of hydroxypropyl and even more of methyl or sulfobutyl groups promoted the BTB inclusion, by expanding the hydrophobic region of the CD cavity, and enhancing the substrate binding through a hydrophobic effect. The stability constant values of the complexes were in the order RAMEB > SBEβCD >> HPβCD > βCD >> HPαCD > αCD >> γCD. The same rank order was observed also as for their solubilizing efficiency towards BTB (Table 1).

### 3.2. Solid-State Characterization of Drug–CD Systems

**Figure 2:**
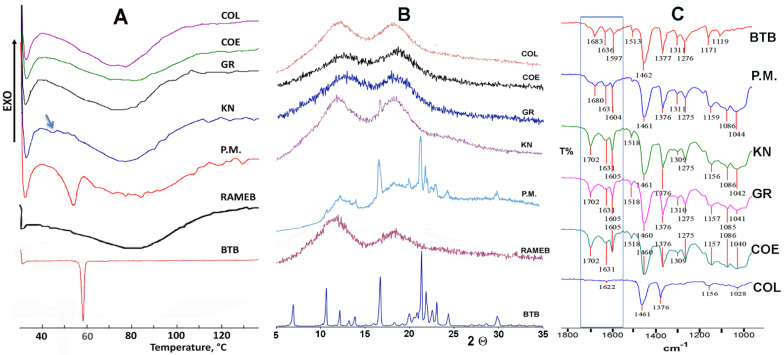
(**A**) DSC curves, (**B**) Xray powder diffraction spectra, and (**C**) FT-IR spectra of pure butamben (BTB) and RAMEB, and of their equimolar solid systems obtained by physical mixing (PM), kneading (KN), co-grinding (GR), coevaporation (COE) and colyophilization (COL).

Based on the above results, RAMEB was chosen for further studies as the best carrier for BTB. The method used for preparing drug–CD solid systems can strongly influence the performance of the obtained products in terms of physicochemical properties and dissolution behavior [42,43]. Therefore, the effectiveness of different techniques (kneading, coevaporation, cogrinding, and freeze-drying) for the preparation of solid equimolar BTB–RAMEB systems was evaluated, in order to individuate the preparation method that is able to give the product the best properties. Simple drug–CD physical mixtures were also prepared for comparison purpose. The solid-state properties of the obtained products were investigated by DSC, XRPD, and FT-IR analyses (Figure 2).

BTB exhibited a thermal behavior typical of a pure, crystalline, anhydrous substance, showing a sharp melting endotherm peaked at 57.7 °C (ΔH 112.2 J/g) (Figure 2A). The thermal profile of RAMEB was instead typical of an amorphous, hydrated substance, as it was characterized by an intense and broad endothermal band, ranged between 60–100 °C, due to the sample dehydration process. The presence of the drug melting peak (even if slightly broadened and shifted at lower temperature, as a consequence of the mixing with the amorphous partner) was evident in the DSC curve of their equimolar PM, thus indicating the absence of appreciable solid-state interactions between the components. On the contrary, only a residual trace of the drug melting endotherm was detected in the KN product (as indicated by the arrow in Figure 2A), and it completely disappeared in the GR, COE, and COL products. This finding, attributable to inclusion complex formation and/or complete BTB amorphization, is certainly indicative of stronger drug-carrier interactions brought about by the preparation techniques utilized.

The XRPD results (Figure 2B) were in full agreement with those of DSC analysis, allowing us to exclude any possible analytical artefact due to the sample heating during the thermal analysis. In fact, the XRPD spectrum of BTB–RAMEB PM showed the presence of the typical crystallinity peaks of the drug, which clearly emerged from the amorphous pattern of the carrier, thus confirming the absence of interactions between the components. Only a residual crystallinity peak at about 17° 2Θ was instead observed in the case of the KN product, whereas a completely amorphous pattern was observed in the case of GR, COE, and COL products, proving the actual drug loss of crystallinity up to complete amorphization and/or inclusion complexation within the cavity of the amorphous partner.

FT-IR analyses (Figure 2C) were also performed as a complement to DSC and XRPD studies. The comparison of the FT-IR spectrum of pure BTB with those of its different equimolar systems with RAMEB can provide some further insight about solid state interactions between the components. Spectra were recorded in the entire 4000–400 cm^−1^ range, but the most important differences were observed in the zone of the carbonyl stretching, as highlighted by the frame in Figure 2C. In fact, spectra of KN, GR and COE products exhibited a shift of the BTB carbonyl band towards higher frequencies (from 1683 to 1702 cm^−1^), as a consequence of its interactions with the RAMEB molecules. Moreover, the complete disappearance of this same band, as well as of those at 1636 and 1597 cm^−1^, was found in the COL product, suggesting a more intimate interaction of BTB with the carrier, and/or a more complete sample amorphization obtained by this preparation technique. On the contrary, all the main BTB absorption bands, including that of the carbonyl band, appeared almost unchanged in the PM spectrum, confirming the absence of interactions between the components after their simple mixing.

### 3.3. Dissolution Studies of BTB–RAMEB Systems

**Figure 3:**
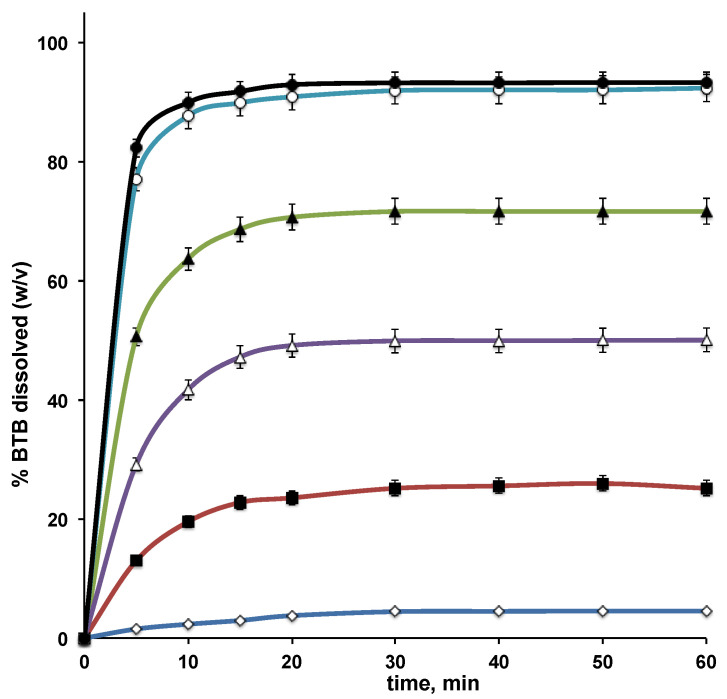
Dissolution curves of butamben (BTB) alone (◊), or from its equimolar physical mixture (∎), kneaded (△), coevaporated (▲), coground (○), and colyophilized (●) products with RAMEB.

The results of dissolution studies of BTB, alone and from its various binary systems with RAMEB, are presented in Figure 3 and Table 2.

As can be observed, all the drug binary systems with RAMEB showed a clear improvement of BTB dissolution properties compared to the plain drug, but clear differences were detected among the products obtained with the different techniques. The increase in BTB dissolution rate observed with the simple PM can be attributed to the wetting effect of RAMEB towards the hydrophobic drug, as well as to the possible “in situ” formation of the complex. A more important enhancement in the drug dissolution behavior, both in terms of dissolution rate and total amount dissolved, was obtained with the KN and COE products, which gave rise to an about 18 and 27 times increase, respectively, of the amount of drug dissolved at 10 min, and to an about 12 and 17 times increase, respectively, of dissolution efficiency at 60 min (DE60). Even better results were obtained with GR and COL products, which allowed us to reach more than 92% of dissolved drug at the end of the test, with an about 38 times increase of the drug percentage dissolved at 10 min and an almost 23 times increase in DE60.

The achieved drug aqueous concentration at equilibrium from these latter two systems was higher than 0.5% *w*/*v*, with a more than 20-fold increase with respect to drug alone. Furthermore, a good stability of the obtained solutions can be expected, since oversaturation levels were not achieved compared to the BTB solubility values resulting from phase-solubility studies (see Figure 1).

These findings confirmed the strong influence of the drug–CD preparation method on the performance of the final product, and they indicate cogrinding and colyophilization as the best techniques for preparing BTB–RAMEB solid systems endowed with optimal dissolution properties. The BTB–RAMEB GR product was finally selected for loading into liposome, as co-grinding is an easier, faster, and less expensive preparation procedure compared to colyophilization.

### 3.4. Development and Characterization of Liposomal Formulations

Numerous scientific findings suggested that the permeation and carrier function of liposomes into the skin can be enhanced by the presence in the lipid bilayer of a charged surfactant, which increases the vesicle fluidity and deformability [22,23]. Moreover, the addition of a cationic surfactant can have the additional advantage to further promote the vesicle uptake into the skin, via ionic interaction with the negatively charged epithelial cells [44]. The absence of cytotoxicity of SA-containing transfersomes was proved in [45]. Previous studies performed by our research group showed that the addition in the vesicle bilayer of the cationic surfactant stearylamine (SA) enabled the increase of therapeutic efficacy, in terms of intensity and duration of action, of liposomal formulations bearing local anesthetics such as benzocaine, butamben, and prilocaine [30,31]. In particular, an optimized liposomal formulation was developed, consisting of a PC:CH:SA mixture at 5.5/1.0/1.5 molar ratios, which was “double-loaded” with 0.7% *w*/*v* free BTB in the lipid phase and 0.3% *w*/*v* BTB as complex with HPβCD in the aqueous phase, which corresponded to the saturation solubility of this complex [30]. In vivo studies on rabbits evidenced a significant increase (*p* < 0.05) in intensity and duration (from 40 to 60 min) of anesthetic effect of the “double-loaded” BTB liposomal formulation with respect to a corresponding formulation loaded with 1% plain BTB in the lipid phase. However, the limited solubility of the BTB–HPβCD complex not only did not allow us to adequately increase the drug concentration in the aqueous phase of the vesicles, but it also gave rise to a reduction of the drug entrapment efficiency with respect to the corresponding liposomes loaded with all of the plain drug (1%) in the lipid bilayer [30].

Therefore, in the present work, phase-solubility studies were performed on a variety of natural and derivative CDs in order to find the most effective complexing and solubilizing agent towards BTB. From these studies, RAMEB emerged as the best partner for the drug, with a more than 40% increase in solubilizing efficiency with respect to the previous partner HPβCD. The greater aqueous solubility of the BTB–RAMEB than the BTB–HPβCD complex enabled us to fully exploit the “double-loading” technique, and to prepare liposomes bearing 0.5% *w*/*v* drug in the aqueous phase—as a hydrosoluble complex with RAMEB—and 0.5% *w*/*v* free drug in the bilayer. In addition, the very higher stability constant of the BTB–RAMEB complex with respect to that of BTB–HPβCD complex (10,460 vs. 1910 M^−1^) should ensure a greater vesicles stability, avoiding any possible problem of competition between the lipid vesicle components and BTB for interaction with the CD [35,36,37]. The safety of RAMEB as excipient in topical formulations, including ocular and nasal ones, was assessed in [46], and the suggested threshold to avoid risks of adverse effects appearance in ocular or nasal formulations is 5% or 10% *w*/*v*, respectively, i.e., amounts clearly higher than that used in the present formulations (<3% *w*/*v*).

Otherwise, in the attempt of even further improving the performance of the liposomal BTB formulation, the effect of adding also sodium cholate (SC) as edge activator was evaluated, considering its proven ability in improving the vesicles’ capacity to penetrate and effectively carry the drug through the skin [24,47]. The absence of cytotoxicity of SC-based transfersomes was shown [48]. Then, a liposomal formulation consisting of a PC:CH:SA:SC mixture at 5.5/1.0/1.5/1.0 molar ratios was also developed.

In order to assess the actual advantages of the “double-loading” approach, both single-loaded (SL) liposomes containing 1% *w*/*v* drug in the lipophilic phase and double-loaded (DL) liposomes containing 0.5% *w*/*v* drug in the aqueous phase, as a hydrosoluble complex with RAMEB, and 0.5% *w*/*v* free drug in the bilayer, were prepared.

The various liposomal formulations were evaluated for mean dimensions, polydispersity index (PDI), Z-potential, deformability, and encapsulation efficiency (Table 3).

No important differences in size or PDI values were observed between double-loaded (DL) and the corresponding single-loaded (SL) liposomes (SL1 vs. DL1, or SL2 vs. DL2) (*p* < 0.05), or even between formulations containing stearylamine (SA) alone or in combination with sodium cholate (SC) (SL1 and DL1 vs. SL2 and DL2) (*p* < 0.05). All the examined liposomal dispersions exhibited good deformability properties, as proven by the values near the unit of the vesicle diameter ratio before and after extrusion, and this property should ensure good penetration and permeability abilities through the biological membranes. Regarding the surface charge of the vesicles, as expected, the presence of the anionic surfactant SC gave rise to a decrease of the positive Z-potential values, with respect to the formulations containing the cationic surfactant SA alone. On the other hand, the slightly lower Z-potential values of SL vesicles with respect to the corresponding DL ones could be due to the presence on their bilayer of more BTB molecules, whose amino group is unionized at physiological pH [49]. Finally, all formulations gave high EE% values, always higher than 90%. However, it is worth highlighting the important advantage achieved with respect to the previously developed liposomal formulation double-loaded with the BTB–HPβCD complex [30]. In fact, in the previous case, due to the limited solubility and stability of the BTB complex with HPβCD, the use of the double-loading approach gave rise to a significant decrease in drug EE% compared to the corresponding formulation containing all the plain drug in the liposomal bilayer [30]. On the contrary, in the present case, in virtue of the greater solubilizing and complexing power of RAMEB towards BTB, no decrease (DL1 vs. SL1) or even an increase (DL2 vs. SL2) in EE% values was observed for DL formulations compared to the corresponding SL formulations. Furthermore, EE% obtained with both DL formulations (93.8% for DL1 and 99.8% for DL2) was clearly higher than the 82% obtained with the previous DL formulation bearing the BTB–HPβCD complex [30]. Finally, the highest EE% value, which was near 100%, was found for the DL2 formulation, containing SA and SC in combination.

**Figure 4:**
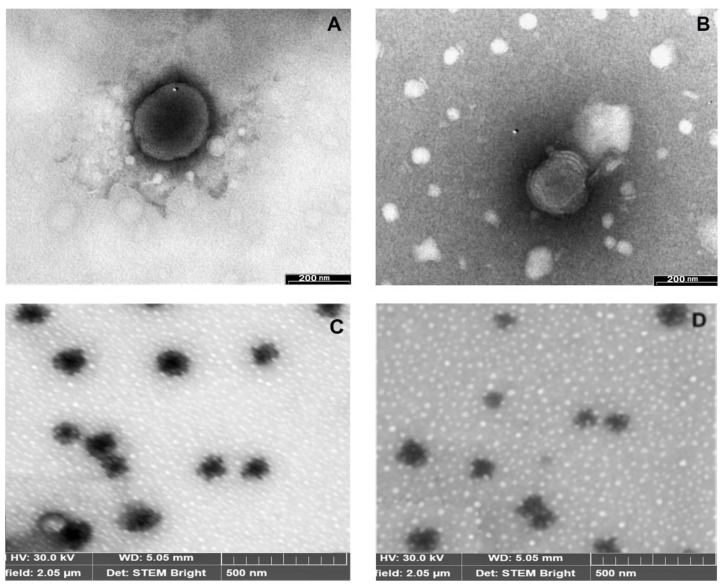
Representative TEM (top) and SEM (bottom) micrographs of SL2 (**A**,**C**) and DL2 (**B**,**D**) liposomal formulations (see Table 3 for liposomal formulation composition).

The TEM and SEM analyses (Figure 4) indicated that all the liposomal vesicles were of homogeneous dimensions and almost spherical shape and presented the typical multilamellar structure. Interestingly, neither appreciable changes in vesicle morphology nor the appearance of aggregation or vesicle destruction phenomena were detected in DL formulations compared to the corresponding SL liposomes, which did not contain RAMEB. This positive outcome may be attributed to the very high affinity of RAMEB for BTB, which avoided possible competition by the lipid components of the bilayer for interaction with this CD and allowed for the formation of stable liposomal vesicles [35,37].

### 3.5. Stability Studies of Liposomal Formulations

A frequent occurrence observed during the storage of liposomal formulations—and is considered indicative of poor stability of the colloidal dispersion—is the increase in vesicle particle size as a consequence of aggregation and/or coalescence phenomena, which can also negatively affect their drug-carrier and drug release properties.

The results of stability studies proved that for all the examined liposomal dispersions, their mean dimensions remained almost unmodified during 3 months storage at 4 °C. In fact, the observed changes in vesicle size were in all cases lower than 10% compared to the values of the corresponding freshly prepared samples. Moreover, the PDI values were stable during that time, indicating the maintenance of homogeneity, and only negligible zeta potential variations were observed, supporting the physical stability of the colloidal dispersion.

Regarding instead the possible problems of drug leakage during storage, EE% values of SL formulations showed a decrease of more than 10% at the end of the storage period, dropping from 92.2 ± 3.8 up to 81.7 ± 2.9, and from 94.6 ± 2.6 up to 83.2 ± 3.1 for SL1 and SL2 formulations, respectively. On the contrary, in the case of DL formulations, the drug leakage phenomenon was clearly more limited, with a decrease of EE% values lower than 5%, passing from 93.8 ± 3.0 to 90.1 ± 2.9 (DL1) and from 99.8 ± 1.3 to 96.2 ±1.6 (DL2). These results proved the better efficiency of the double-loading technique in reducing the drug leakage from the liposomal vesicles, given that the portion of drug present in the internal aqueous phase as a hydrophilic CD complex is less prone to be prematurely released compared to the free drug present in the vesicle bilayer.

### 3.6. In Vitro Drug Permeation Studies

**Figure 5:**
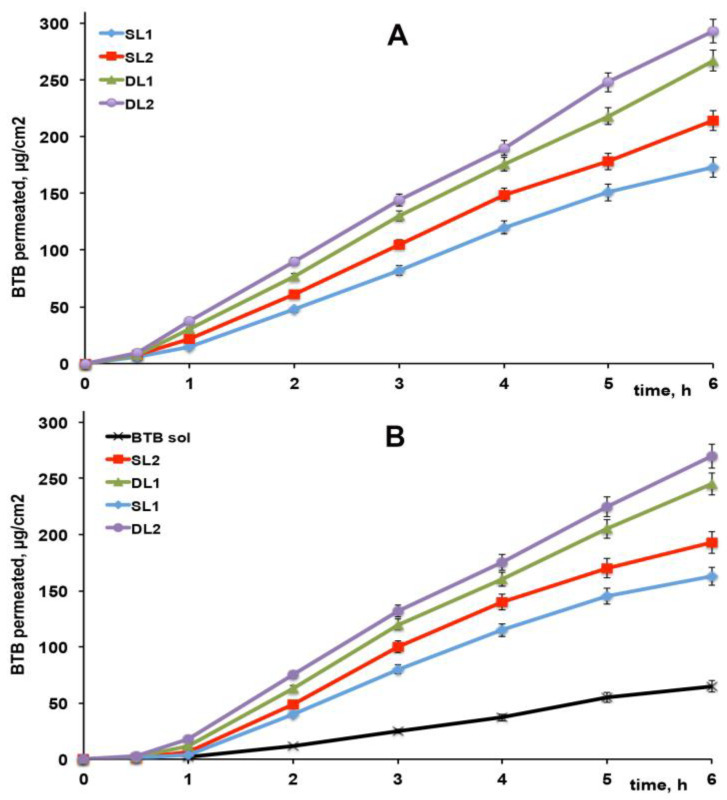
Permeation profiles through excised rabbit ear skin of butamben (BTB) from 1% *w*/*v* drug-loaded liposomal dispersions as such (**A**) or dispersed (50:50 *w*/*w*) in an aqueous Carbopol gel (**B**) (see Table 3 for liposomal formulations composition).

The drug apparent permeability coefficient (Kp), obtained by permeation studies across excised rabbit skin of BTB from the different liposomal dispersions either as such or formulated as Carbopol gel, are reported in Table 4; the related drug permeation profiles are shown in Figure 5A,B with the curve obtained from the drug solution in Carbopol gel.

An initial lag-phase was observed, attributed to the time necessary to saturate the skin membrane; it was slightly longer for gel formulations, probably due to the presence of the polymeric network, which decreased somewhat the drug diffusion.

All liposomal formulations, both as such (Figure 5A) or formulated as gel (Figure 5B), showed clearly better drug permeation properties (*p* < 0.001) than the drug solution, confirming the skin penetration ability and permeation-enhancing properties of elastic liposomal vesicles on drug delivery [22,23,24,25,26,27]. Gel liposomal formulations showed a slight, even though not significant (*p* > 0.05) reduction in the drug permeation rate with respect to the corresponding liposomal dispersions, which could be attributed, as in the case of the initially longer lag-time phase, to the presence of the gel network, which slowed down the drug diffusion rate.

As can be observed, DL liposomes exploiting the “drug-in CD- in liposome” approach showed significantly better (*p* < 0.05) drug permeation profiles than the corresponding SL ones. A role of RAMEB as a skin permeation enhancer could be hypothesized to explain this result. Similar findings have been reported in the case of microemulsions or suspensions, where the RAMEB addition to the formulations allowed for a significant improvement in drug permeation properties through excised animal membranes, attributed to the RAMEB’s ability to reversibly remove lipids form the stratum corneum, thus temporarily reducing its barrier effect [29,50].

Moreover, a better performance of DL2 and SL2 liposomal formulations than the corresponding DL1 and SL1 ones was observed, even though it was of borderline statistical significance (*p* ≈ 0.05). This effect could be explained with the joined presence in the first ones of SA and SC, which probably enabled a more efficient drug permeation.

### 3.7. In Vivo Studies

**Figure 6:**
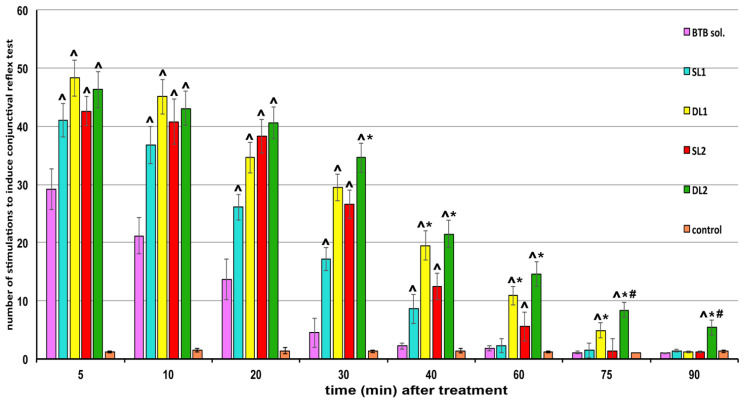
Effect induced on the rabbit conjunctival reflex test by different Carbopol gel formulations containing 0.5% butamben (BTB) as hydroalcoholic solution or single-loaded (SL) or double-loaded (DL) liposomal formulations (see Table 3 for liposomal formulations composition). Six rabbits per group. Mean of six experiments. The control consisted of a Carbopol gel containing the corresponding formulation without drug. Note: ˆ *p* < 0.05 vs. drug solution; * *p* < 0.05 vs. SL formulation; ^#^ *p* < 0.05 vs. DL1 formulation.

The results of in vivo studies, presented in Figure 6, proved that all the liposomal gel formulations gave rise to a significant (*p* << 0.05) increase in both intensity and duration of action in comparison with the gel containing the drug hydroalcoholic solution, whose anesthetic effect was significantly different from the respective blank control for only 30 min. Both the drug loading mode, as well as the presence or absence in the vesicle bilayer of SC together with SA, played a role in the performance of the liposomal formulations. In fact, formulations exploiting the double-loading approach showed a duration of action significantly longer (*p* < 0.05) than the corresponding single-loaded formulations, allowing for prolonging the anesthetic effect up to 75 min (DL1) or even up to 90 min (DL2), with respect to 40 min and 60 min obtained with the corresponding SL1 and SL2 formulations. The better performance of DL formulations than the SL ones can be explained by the combined presence of a free drug fraction in the external bilayer, which provided a rapid initial effect, and of a drug fraction in the internal vesicle core, in the form of a complex with RAMEB, which ensured a more prolonged drug release profile.

Moreover, the anesthetic effect provided by DL1 formulation was more prolonged even than that obtained with the previously developed DL formulation of similar composition, but containing BTB as HPβCD complex (75 vs. 60 min) [29], thus confirming the important role played by the CD type used for drug complexation in affecting its release properties from the liposomal vesicles [16,35,36]. This result can be in fact ascribed to both the higher solubilizing effect of RAMEB towards BTB, which allowed us to increase the drug loading in the aqueous core of the vesicles, as well as to achieve a higher stability of the BTB–RAMEB complex, which in turn enabled a better control of the drug release.

Regarding instead the greater effectiveness of formulations containing SA in combination with SC (DL2 vs. DL1 and SL2 vs. SL1), it can be explained by the enhancer effect of this last edge activator, which probably favored a better penetration through the skin of the liposomal vesicles [51]. On the other hand, a possible synergistic effect between the cationic surfactant SA and the anionic surfactant SC in increasing skin permeability could be also hypothesized, as observed by other authors [52].

## 4. Conclusions

A new effective liposomal formulation of BTB endowed with enhanced skin delivery and increased duration of the drug anesthetic effect was successfully developed. RAMEB, selected by preliminary phase-solubility studies as the best complexing agent for BTB, was utilized to develop deformable liposomes double-loaded with 0.5% plain drug in the vesicle external bilayer and 0.5% drug as a hydrophilic complex with RAMEB in the internal aqueous core.

The use of RAMEB for BTB complexation revealed to be essential for improving the performance of the liposomal formulations, which showed to be clearly superior with respect to a previously developed formulation based on the HPβCD–BTB complex [30], thus evidencing the importance of the proper choice of the CD type used for drug complexation in order to take maximum advantage of the “double loading” strategy. In fact, the greater solubility and the very higher stability of the BTB–RAMEB than the BTB–HPβCD complex made it possible not only to increase the drug entrapment efficiency of the vesicles but also to obtain a more controlled and prolonged drug release, as proven by the significant increase in duration of BTB anesthetic effect obtained in in vivo experiments.

The superior properties of double-loaded (DL) liposomes than the corresponding single-loaded (SL) ones, particularly in terms of greater stability towards drug leakage during storage and of better control of drug release, with a consequent more prolonged drug anesthetic effect in vivo, was also proven. Moreover, the influence of the edge activator type on the performance of deformable liposomes was confirmed. In particular, DL-liposomes containing the combination of SA and SC emerged as the best formulation, showing not only the highest entrapment efficiency (99%) but also the longest duration of drug anesthetic effect in vivo (90 min).

Finally, a strong correlation between in vitro BTB permeation experiments through excised rabbit ear skin and in vivo anesthetic efficacy was found, indicating that in vitro studies could be useful in preclinical tests for selecting the most efficient formulation to subject to in vivo studies.

## Figures and Tables

**Table 1:** Stability constants (K_1:1_) and solubilizing efficiency of the different examined CDs towards butamben.

Type of CD	K_1:1_, M^−1^	Solubilizing Efficiency at 12.5 mM CD *	Solubilizing Efficiency at 25 mM CD *
αCd	480 ± 30	5.7	9.2
βCD	1390 ± 90	8.7	--- ^§^
γCD	80 ± 10	1.2	1.2
HPαCD	540 ± 40	6.1	10.5
HPβCD	1910 ± 100	10.2	19.2
RAMEB	10460 ± 220	13.0	27.2
SBEβCD	3590 ± 130	11.9	23.6

* ratio between drug solubility in the presence of a given CD concentration, and solubility of drug alone (0.86 mM); ^§^ not determinable (βCD solubility < 25 mM).

**Table 2:** Percentage dissolved at 10 min (PD10) and dissolution efficiency at 60 min (DE60) of butamben (BTB) alone and from its equimolar physical mixture (PM), kneaded (KN), coevaporated (COE), coground (GR), and colyophilized (COL) products with RAMEB.

Sample	PD10	DE60
BTB	2.3	3.7
BTB–RAMEB PM	19.5	22.4
BTB–RAMEB KN	41.7	45.1
BTB–RAMEB COE	63.3	65.9
BTB–RAMEB GR	87.7	86.3
BTB–RAMEB COL	89.9	88.0

**Table 3:** Composition, particle size, polydispersity index (PDI), Z-potential, deformability and encapsulation efficiency (EE%) of butamben liposomes single-loaded (SL) with 1% *w*/*v* free drug in the lipid phase, or double-loaded (DL) with 0.5% *w*/*v* free drug in the lipid phase and 0.5% *w*/*v* in the aqueous phase as RAMEB complex.

Formul. Code	Bilayer Composition (Molar Ratios)				Drug Loading Mode	Vesicle Size (nm ± SD)	PDI	Z-Pot. (mV ± SD)	Deformability	EE%
	PC	CH	SA	SC						
SL1	5.5	1.0	1.5		SL	240 ± 65	0.22	+30.2 ± 3.9	1.10 ± 0.02	92.2 ± 3.8
DL1	5.5	1.0	1.5		DL	280 ± 60	0.24	+39.8 ± 2.8	1.08 ± 0.03	93.8 ± 3.0
SL2	5.5	1.0	1.5	1.0	SL	260 ± 40	0.23	+5.1 ±0.5	1.05 ± 0.04	94.6 ± 2.6
DL2	5.5	1.0	1.5	1.0	DL	290 ± 56	0.25	+12.1 ± 0.5	1.03 ± 0.03	99.8 ± 1.3

**Table 4:** Permeability coefficient (Kp) of butamben (BTB) from the different formulations (mean ± SD, *n* = 6).

Formulation	Kp (cm/h)
SL1	0.0433 ± 0.0038
SL2	0.0516 ± 0.0047
DL1	0.0630 ± 0.0054
DL2	0.0672 ± 0.0057
SL1 in gel	0.0426 ± 0.0034
SL2 in gel	0.0491 ± 0.0041
DL1 in gel	0.0601 ± 0.0050
DL2 in gel	0.0652 ± 0.0054
BTB solution in gel	0.0181 ± 0.0015

## Data Availability

Not applicable.
